# Predictors of HIV/AIDS Related Ocular Manifestations among HIV/AIDS Patients in Felege Hiwot Referral Hospital, Northwest Ethiopia

**DOI:** 10.1155/2015/965627

**Published:** 2015-04-27

**Authors:** Guadie Sharew, Muluken Azage

**Affiliations:** ^1^Department of Ophthalmology, College of Medicine and Health Sciences, Bahir Dar University, P.O. Box 79, Bahir Dar, Ethiopia; ^2^Department of Public Health, College of Medicine and Health Sciences, Bahir Dar University, P.O. Box 79, Bahir Dar, Ethiopia

## Abstract

*Background*. Ocular manifestations in people living with HIV/AIDS are varied and affect almost all the structures of eye leading to visual impairment or blindness. Therefore, the aim of this study was to identify the predictors of HIV related ocular manifestation among ART clinic clients. *Methods*. Institution based cross-sectional study was employed among ART clients at Felege Hiwot referral hospital, northwest Ethiopia. The study was conducted from 1 January 2013 to 30 January 2013. A total of 369 systematically and randomly selected clients were included in the study. Data were collected using structured questionnaires and ophthalmologic clinical examination. Data were entered and analyzed using SPSS version 16.0. Binary and multivariable logistic regression analyses were computed to identify independent predictors of HIV related ocular manifestation. *Results*. Twenty-five percent (25.7%) of HIV patients had ocular manifestations. The three most frequent signs were Squamoid Conjuctival growth (26.9%), ophthalmic herpes zoster (22.1%), and Bacterial Conjuctivitis (17.2%). History of eye problem, CD_4_ count, and visual acuity of the eye were the predictors of HIV related ocular manifestation. *Conclusion*. In this study, a higher proportion of ocular manifestations were detected in HIV/AIDS patients. Visual acuity and CD_4_ counts were the independent predictors of ocular manifestations. This finding gives an insight for policy makers and concerned body to integrate ophthalmic examination in ART clinics to improve the health condition of HIV/ADIS patients.

## 1. Introduction

Ocular manifestations among people living with Human Immunodeficiency Virus (HIV)/Acquired Immunodeficiency Syndrome (AIDS) are varied and affect almost all the structures of the eye. Approximately 70–80% of HIV-infected patients are expected and treated for HIV related eye disorder during the course of their illness [1]. The life time risk of having at least one abnormal ocular lesion among HIV patients ranges from 52% to 100% [2–4]. Sub-Saharan African (SSA) countries are disproportionately hit by the virus accounting for the largest burden of HIV/AIDS; by 2008, about 22.4 million (20.8–24.1) people were living in the region [5]. Ethiopia is one of those countries affected by HIV with 1.4% of its more than 80 million population infected with HIV [6].

HIV affects the immune system that the virus can either directly inflict damage to the organs of the body by itself and/or make the organs vulnerable to many opportunistic pathogens and diseases. There are wide arrays of diseases affecting the eyes of people living with HIV/AIDS that can occur at any time along the natural course of the disease. HIV related ophthalmic disorders occur due to several causes like opportunistic infections, vascular abnormalities, neoplasm, and drug induced and neuroophthalmic lesions. Among them, opportunistic infections are the major cause of morbidity and the most devastating ophthalmic disorder in people with AIDS [7, 8]. HIV-cytomegalovirus (CMV) coinfection occurs in 75–85% of patients, more than half of which develop CMV retinitis which is vision threatening. Despite this high incidence, difficulties concerning the therapeutic approach and the result are relatively unsatisfactory even with the highly active antiretroviral therapy (HAART) [9]. On top of this, there is a 63% risk of immune recovery uveitis (IRU) in patients with regressed CMV retinitis [10, 11].

In SSA, the prevalence of ocular disease in HIV-positive individuals was between 30% and 45% [12, 13]. The spectrum of ocular diseases in HIV-infected patients in developing countries is different from that in developed countries [4, 14]. Most notably, while antibodies against CMV are detectable in 90% of people living with HIV/ADIS, CMV retinitis was rare (less than 5%) in AIDS patients in developing countries [15, 16]. Ocular manifestations affecting only one eye like herpes zoster ophthalmicus and conjunctival squamous cell carcinoma are, however, relatively common in developing countries [15, 16]. The reasons for such variations in the distribution were presumed to be the early and high mortality rate in people living with HIV/AIDS in developing countries and possibly differences in HIV subtype, race, and the influence of comorbid diseases.

Few studies in Ethiopia showed that the occurrence of at least one ocular manifestation was estimated between 32–60% among HIV/AIDS patients [17, 18]. CD_4_ count was mentioned as predictor of ocular infections in patients who are living with HIV. Many findings claimed that CD_4_^+^ count less than 100 cells/*μ*L was consistently associated with ocular manifestations in HIV-positive patients [17–23].

In Ethiopia, only few studies were done with patterns of ocular manifestation among HIV patients. However, there is scanty of credible evidence on predictors of ocular manifestation among these vulnerable groups and the existing findings are not exhaustively included in the independent variables such as WHO clinical stage of HIV/AIDS. Data on ocular manifestation among HIV patients and its relation with CD_4_ count will help policy makers, other concerned bodies, and clinicians to integrate new strategy with ART strategies and provide evidence based clinical practice. Therefore, the purpose of this study was to identify factors associated with ocular manifestations among HIV/AIDS patients in Felege Hiwot referral hospital.

## 2. Methods and Materials

### 2.1. Study Design and Setting

Institution based cross-sectional study was employed among ART clients at Felege Hiwot referral hospital, Amhara region, northwest Ethiopia. The study was conducted from 1 January 2013 to 30 January 2013. The ART clinic at Felege Hiwot referral hospital is one of the largest in Amhara region, which has been serving more than 400 patients per week. All ART clients during the study period at Felege Hiwot hospital were the source population.

#### 2.1.1. Sample Size and Sampling Technique

The sample size was determined using single proportion formula with the following assumptions: estimated proportion of ocular manifestation of 60% (*p* = 0.60) [18], 95% CI (*Z*_*α*/2_ = 1.96), and a 5% margin of error (*d* = 0.05). The final sample size was 388 by including 5% nonresponse rate. Study participants were selected using a systematic random sampling technique in the ART clinic of the Felege Hiwot hospital. A cue note was attached on identity card to avoid double counting during the study period.

#### 2.1.2. Data Collection Tools

Structured questionnaire was used to collect sociodemographic data and volunteer participants had undergone ophthalmologic examination for possible HIV related ophthalmologic manifestations. The findings from the eye clinical examinations and their recent CD_4_ counts were recorded using a prepared data recording format sheet at ART clinic. For patients who did not have a CD_4_ count within the last three months, CD_4_ count was done during the study. Thorough examination of the eyes, including external eye inspection, ocular motility, and slit lamp examination of the external eye, as well as pupils, lens, and the anterior vitreous was done for all the patients. Posterior segment was evaluated by dilating pupils and examining using indirect Ophthalmoscope and/or 90D Volk lens. Squamoid type conjunctival growth was clinically defined as a suspicious conjunctival mass on slit lump exam though histology was not done due to lack of facility during the study period. Blindness was defined as visual acuity (VA) of less than counting finger (CF) at three meters with the better eye. Visual acuity of less than 6/18 was defined as visual impairment.

#### 2.1.3. Data Quality

Questionnaire was pretested to ensure whether the study participants understood what the investigators intended to know. The questionnaire was modified based on the pertest result. Second opinion was sought from another ophthalmologist as a means of quality control specially in cases of eyes with squamoid growth. Data collection procedure and data collection format were discussed with data collectors. Moreover, proper functionality of materials used for clinical eye examination was checked daily before the examination.

#### 2.1.4. Data Analysis

Questionnaires were coded and data were entered and analyzed using SPSS software version 16. Descriptive statistics and binary logistic regression analyses were carried out to describe variables and to identify factors associated with ocular manifestation, respectively. Backward stepwise multivariable logistic regression analysis was used to identify multicollinearity free predictors of ocular manifestation. Crude and adjusted odds ratios with 95% confidence intervals were calculated as cut-off point to identify the predictors of ocular manifestations.

#### 2.1.5. Ethical Statement

Ethical approval was obtained from the Ethical Clearance Committee of Bahir Dar University. Permission was taken from the Regional Health Bureau as well from Hospital administration. Informed and written consents were obtained from the study participants. Privacy and confidentiality were maintained throughout the study period; each questionnaire was number-coded without any personal identification. Intervention measures such as health education, detailed counselling, and medical therapy were provided whenever necessary during data collection. Those who needed surgical intervention and further eye follow-up were referred to the hospital's Eye Unit. All eye drops used for examination were all the ones registered by Drug Administration and Control Authority (DACA) for use in Ethiopia. The effects of drugs used were explained to the patients and data collected were kept confidential.

## 3. Results

A total of 370 clients were included in the study with the response rate of 95%. One hundred and thirty-nine (37.8%) were males. The mean (SD) age of the respondents was 32.5 (8.7) years. Two hundred and thirty (62.3%) were urban residents and 29.7% were daily laborers. The majority (93.8%) of the study participants were Orthodox Christian, 19.5% were single, and one-third of the study participants were unable to read and write (Table 1).

### 3.1. Clinical Characteristics of HIV/AIDS Patients

A majority, 267 (74.1%), of participants were on WHO clinical stage III. Ninety-eight percent (98%) of the study participants were on ART at the time of the study and 37% of the study participants have taken ART for more than five years. Seventy-nine percent of the study participants had a recent CD_4_ count of 200 and above. About 25.4% of the participants had a history of eye problem. Almost ninety percent of the study participants (89.2%) were found to have normal visual acuity and 25.7% of the study participants had at least one sign of the ocular manifestation (Table 2).

### 3.2. Type of HIV/AIDS Associated Ocular Manifestations

The most frequent ocular manifestations were squamoid conjunctival growth (26.9%) and ophthalmic herpes zoster (22.1%). Molluscum contagiosum (0.7%) and vernal conjunctivitis (0.7%) were among the less frequent ocular manifestations (Table 3).

### 3.3. Predictors of HIV/AIDS Related Ocular Manifestations

In this study, history of eye problem, CD_4_ count, and visual acuity of the eye were factors associated with HIV/AIDS related ocular manifestation. After controlling confounding factors and interaction effect of the variables using backward stepwise regression, history of eye problem, CD_4_ count, and visual acuity of the eye remained statistically significant predictors of HIV/AIDS related ocular manifestation among HIV/AIDS patients (Table 4).

Therefore, in this study, those who reported to have a history of eye problem {AOR: 11.26, 95% CI: (5.87, 21.68)} were 11 times more likely to develop ocular manifestation compared to clients who did not report history of eye problem. Those clients who had a recent CD_4_ count of <200 {AOR: 7.42, 95% CI: (3.62, 15.21)} were 7.42 times more likely to develop ocular manifestation than those clients who had a CD_4_ count of >200. Similarly, those clients who had a visual impairment {AOR: 3.83, 95% CI: (1.38, 10.62)} were 3.83 times more likely to have HIV/AIDS related ocular manifestation than those who had normal visual acuity (Table 4).

## 4. Discussion 

In this study, the prevalence of ocular manifestation was 25.7%, which is consistent with recent studies done in Jimma in 2009 (25.3%) [24] and in Gondar in 2010 (21.4%), Ethiopia [25]. However, it is much lower than the previous study conducted at Gondar University Hospital in 2004 which was 60% [18]. This discrepancy could be due to the introduction and use of HARRT in ART clinics and difference in the nature of the study population. At Gondar referral hospital, the study was carried out on admitted patients in hospital with a medical problem and on those who came to the eye clinic with ocular complaint, while our study included randomly selected individuals in ART clinic.

The most frequent ocular manifestations were squamoid type conjunctival growth (26.9%) and ophthalmic herpes zoster (22.1%), bacterial conjunctivitis (17.2%), and microvaculopathy (RM) (15.2%) which is consistent with findings in other studies done in Gondar and Jimma, Ethiopia [24, 25]. In studies done in other African countries, the proportion of microvaculopathy was reported in the range of 10–42% [26, 27] and, moreover, in India [28] it was reported that 50% of the study participants had microvaculopathy which is higher than the results of this study. This difference may be due to the potential genetic or other differences in the study populations in ART clinics.

Three-fourth of the study participants (77.9%) in this study were in WHO stages III and IV, which is a lower figure from the study done in Gondar referral hospital (90%) [18]. The difference is due to the fact that the Gondar study participants were mainly critically ill and admitted patients while ours are OPD ART followers who are potentially in a better health status.

It was found that a higher proportion (58%) of those patients whose CD_4_ count <200 were more likely to have ocular manifestation than those patients whose CD_4_ count >200 (42%). This finding is consistent with the general facts of immunosuppression and is consistent with the results of the other studies in Ethiopia (Jimma) [24] and India [28]. Similarly, we found that lower CD_4_ count (of less than 200) was statistically significant predictor of ocular manifestations which is consistent with the study done in Jimma [24].

The proportion of patients with low duration on ART had a high occurrence of ocular manifestations compared to their counterparts because ART drugs may have a role in increasing the CD_4_ counts and boosting the immunity status of patients which reduce the occurrence of opportunistic infections. Similarly, in this study we found that the proportion of ocular manifestations was higher in patients with advanced stage of the disease (stages III and IV). The above finding is consistent with other studies [24].

History of eye problem was also statistically significantly associated with ocular manifestations in this study which is consistent with the study done in Gondar [18]. Moreover, multivariate logistic regression analysis showed that an impaired visual acuity (less than normal V/A) was found to be an independent predictor for ocular manifestations which is consistent with the finding in Gondar, Ethiopia [18].

## 5. Conclusions 

The proportion of ocular manifestations among HIV/AIDS patients was high in this study. Higher proportions of ocular manifestations were detected in HIV/AIDS patients with low CD_4_ counts and low duration on ART and in advanced stage of the diseases. Low visual acuity, history of eye problem, and CD_4_ counts were the independent predictors of ocular manifestations. The routine current practice at ART clinics used CD_4_ counts as a reminder for occurrence of opportunistic infections. It would be advisable to use ocular manifestations as indicators among the many OI for the patient to be aware. Moreover, this finding gives an insight for policy makers and concerned body to integrate ophthalmic examination in ART clinics to improve the health condition of HIV/ADIS patients, if the patient may late to present to eye clinic for intervention. During data collection those who had CMV retinitis were referred to eye department, but it was not possible to find any treatment options for CMV neither in the hospital pharmacy nor in the town. So this finding could fill the information gap and it may also give a clue for suppliers to start providing some treatment or medications for ocular opportunistic infections.

## Figures and Tables

**Table 1:** Sociodemographic characteristics of HIV/AIDS patients in ART clinic of Felege Hiwot referral hospital, northwest Ethiopia, January 2013.

Variable	Total study subject	
	Number (370)	Percent
Sex		
Male	140	37.8
Female	230	62.2
Age		
<28	90	24.3
28–30	94	25.4
31–37	88	23.8
>37	98	26.5
Occupational status		
Daily labourer	110	29.7
Student	14	3.8
Government employee	88	23.8
Housewife	45	12.2
Merchant	28	7.6
Unemployed	37	10.0
Self-employee	32	8.6
Others	16	4.3
Residence		
Urban	230	62.2
Rural	140	37.8
Religion		
Orthodox	346	93.8
Muslim	20	5.4
Protestant	3	0.8
Marital status		
Single	72	19.5
Married	187	50.5
Separated	44	11.9
Widowed	7	1.9
Divorced	60	16.2
Education status		
Uneducated	122	33.1
Read and write	24	6.5
1 to 6 grade	58	15.7
7 to 12 grade	100	27.1
Above 12 grade	66	17.8

**Table 2:** Clinical characteristics of HIV/AIDS patients in ART clinic of Felege Hiwot referral hospital, northwest Ethiopia, January 2013.

Variable	Number	Percent
WHO clinical stage of HIV/AIDS		
Stage I	31	8.4
Stage II	51	13.8
Stage III	274	74.1
Stage IV	14	3.8
Duration of HIV in years		
<5 years	185	50.0
	185	50.0
Duration of ART in years		
	233	63.0
	137	37.0
CD_4_ count		
	77	20.8
	293	79.2
On ART		
Yes	363	98.1
No	7	1.9
History of eye problem		
Yes	94	25.4
No	276	74.6
Visual acuity of both eyes		
Normal	330	89.2
Visual impairment	40	11.1
At least one sign of ocular manifestation (*n* = 369)		
Yes	95	25.7
No	275	74.3

**Table 3:** Pattern of HIV/AIDS associated ocular manifestations in ART clinic of Felege Hiwot referral hospital, northwest Ethiopia, January 2012.

Ocular manifestation	Frequency	Percentage
Squamoid conjunctival growth	39	26.9
Ophthalmic herpes zoster	32	22.1
Bacterial conjunctivitis	25	17.2
Microvasculopathy (RM) (early retinal discharge)	22	15.2
Seborrheic blepharitis	9	6.2
Cytomegalovirus (CMV) retinitis	7	4.8
Uveitis	4	2.8
Neuroophthalmic disorders^∗^	3	2.1
Xanthelasma	2	1.4
Molluscum contagiosum	1	0.7
Vernal conjunctivitis	1	0.7

^∗^Optic atrophy and papilledema were the cases.

**Table 4:** Regression analysis for ocular manifestations among HIV/AIDS patients in ART clinic of Felege Hiwot referral hospital, northwest Ethiopia, January 2012.

Variable	Ocular manifestation		COR (95% CI)	AOR (95% CI)
	Yes	No		
Sex				
Male	38	102	1.13 (0.70–1.83)	
Female	57	173	1.00	
Age				
	25	66	1.00	
28–30	18	76	0.63 (0.81–3.24)	
31–37	21	66	0.73 (0.59–2.25)	
	31	67	1.22 (0.46–1.61)	
Residence				
Urban	59	171	1.00	
Rural	36	104	1.00 (0.61–1.61)	
Religion				
Orthodox	89	258	0.69 (0.37–2.93)	
Muslim	5	15	0.67 (0.06–7.70)	
Protestant	1	2	1.00	
Marital status				
Single	14	58	1.00	
Married	49	138	1.47 (0.33–1.24)	
Widowed	15	29	2.14 (0.24–1.38)	
Separated	1	6	0.69 (0.16–13.02)	
Divorced	16	44	1.50 (0.29–2.50)	
Education status				
Uneducated	34	88	1.00	
Read and write	8	16	1.29 (0.30–1.97)	
1 to 6 grade	13	45	0.75 (0.64–2.78)	
7 to 12 grade	23	77	0.77 (0.70–2.38)	
Above 12 grade	17	49	0.78 (0.55–2.15)	
WHO clinical stage				
Stage I	9	22	1.00	
Stage II	10	41	0.60 (0.21, 1.69)	
Stage III	69	205	0.82 (0.36, 1.87)	
Stage IV	7	7	2.44 (0.59, 4.74)	
Duration of HIV				
	53	132	1.37 (0.86, 2.19)	
	42	143	1.00	
History of eye problem				
Yes	66	28	20.08 (11.17–36.07)^∗∗^	11.26 (5.87, 21.68)^∗∗^
No	29	247	1.00	1.00
CD_4_ count				
	55	22	15.81 (8.71, 28.71)^∗∗^	7.42 (3.62, 15.21)^∗∗^
	40	253	1.00	1.00
On ART				
Yes	94	269	1.00	
No	1	6	0.48 (0.06–4.01)	
Visual acuity of both eyes				
Normal	64	266	1.00	1.00
Visual impairment	31	9	14.32 (6.49–31.57)^∗∗^	3.83 (1.38, 10.62)^∗^

^∗^ *p* < 0.05, ^∗∗^ *p* < 0.001.

## Abbreviations

AIDS: Acquired immunodeficiency syndrome
AOR: Adjusted odds ratio
ART: Antiretroviral therapy
CD: Cluster of differentiation
CI: Confidence interval
CMV: Cytomegalovirus
COR: Crude odds ratio
HAART: Highly active antiretroviral therapy
HIV: Human immunodeficiency virus
HZO: Herpes zoster ophthalmicus
SPSS: Statistical packages for Social Science
SSA: Sub-Saharan African countries
OI: Opportunistic infections.
